# Glyceroglycolipid Metabolism Regulations under Phosphate Starvation Revealed by Transcriptome Analysis in *Synechococcus elongatus* PCC 7942

**DOI:** 10.3390/md18070360

**Published:** 2020-07-13

**Authors:** Xinrui Xu, Xiaoling Miao

**Affiliations:** 1 State Key Laboratory of Microbial Metabolism, School of Life Sciences and Biotechnology, Shanghai Jiao Tong University, 800 Dongchuan Road, Shanghai 200240, China; xuxinrui0603@163.com; 2 Joint International Research Laboratory of Metabolic & Developmental Sciences, Shanghai Jiao Tong University, Shanghai 200240, China; 3 Biomass Energy Research Center, Shanghai Jiao Tong University, Shanghai 200240, China

**Keywords:** glyceroglycolipid metabolism, phosphate starvation, transcriptome, glyceroglycolipid homeostasis

## Abstract

Glyceroglycolipids, abundant in cyanobacteria’s photosynthetic membranes, present bioactivities and pharmacological activities, and can be widely used in the pharmaceutical industry. Environmental factors could alter the contents and compositions of cyanobacteria glyceroglycolipids, but the regulation mechanism remains unclear. Therefore, the glyceroglycolipids contents and the transcriptome in *Synechococcus elongatus* PCC 7942 were analyzed under phosphate starvation. Under phosphate starvation, the decrease of monogalactosyl diacylglycerol (MGDG) and increases of digalactosyl diacylglycerol (DGDG) and sulfoquinovosyl diacylglycerol (SQDG) led to a decrease in the MGDG/DGDG ratio, from 4:1 to 5:3, after 12 days of cultivation. However, UDP–sulfoquinovose synthase gene *sqdB*, and the SQDG synthase gene *sqdX*, were down-regulated, and the decreased MGDG/DGDG ratio was later increased back to 2:1 after 15 days of cultivation, suggesting the regulation of glyceroglycolipids on day 12 was based on the MGDG/DGDG ratio maintaining glyceroglycolipid homeostasis. There are 12 differentially expressed transcriptional regulators that could be potential candidates related to glyceroglycolipid regulation, according to the transcriptome analysis. The transcriptome analysis also suggested post-transcriptional or post-translational regulations in glyceroglycolipid synthesis. This study provides further insights into glyceroglycolipid metabolism, as well as the scientific basis for glyceroglycolipid synthesis optimization and cyanobacteria glyceroglycolipids utilization via metabolic engineering.

## 1. Introduction

Glyceroglycolipids are widely distributed in plants, microalgae and cyanobacteria. Monogalactosyl diacylglycerol (MGDG), digalactosyl diacylglycerol (DGDG) and sulfoquinovosyl diacylglycerol (SQDG) are the three main glyceroglycolipids in the photosynthetic membrane, which are essential for photosynthesis [1]. In cyanobacteria, about 50% of the photosynthetic membrane lipids are MGDG, 20% are DGDG and 16% are SQDG [2]. Glyceroglycolipids present both bioactivities and pharmacological activities, and can be widely used in the pharmaceutical industry [3]. Microalgae and cyanobacteria are competitive sources of glyceroglycolipids because of their abundant glyceroglycolipids, their simple cell structure and their eco-friendly characteristic. Many glyceroglycolipids with pharmaceutical value have been isolated from microalgae and cyanobacteria. MGDG with pro-apoptotic activity is extracted from *Phaeodactylum tricornutum* [4]. MGDG from *Tetraselmis chuii* and *Nannochloropsis granulate* have anti-inflammatory activities [5]. MGDG and DGDG from *Chlorella Vulgaris* [6] and *Phormidium tenue* [7] also present anti-tumor activities. In addition, SQDG, with eukaryotic DNA polymerase inhibitory activity, has been extracted from *Gigartina tenella* [8]. The SQDG isolated from the cyanobacteria *Lyngbya lagerheimii* [9] and *Phormidium tenue* [10] have AIDS-antiviral activities. Recent studies have also identified the immuno-stimulatory activity and the potential against Alzheimer’s disease of SQDG derived from microalgae [5].

Environmental factors directly influence the accumulation of many metabolites. Phosphate—the main ingredient of nucleic acid, protein and phospholipids—is an essential element in organism growth. However, phosphate is often limited in natural environments [11]. To cope with phosphate limitation, organisms have acquired different strategies, including ultrastructural rearrangements, C reallocation, transcriptome reprograming, and metabolome and lipid remodeling [11]. Glyceroglycolipid accumulation could be strongly regulated by phosphate concentration. In plants, the synthesis of some kinds of glyceroglycolipids (like DGDG and SQDG) will be induced when phosphate is lacking during cultivation, in order to supplement the shortage of phospholipids so as to maintain both the structures and functions of membranes [12,13]. A similar phenomenon was also reported in the cyanobacteria *Synechococcus elongatus* PCC 7942, wherein phosphate starvation resulted in a decrease in phospholipid and an increase in SQDG [14]. In *Chlamydomonas nivalis*, DGDG increased while MGDG decreased under phosphate deprivation [15]. In addition, an increase in total glyceroglycolipids is common in microalgae and cyanobacteria when exposed to a phosphate deficiency condition [16,17,18]. 

Glyceroglycolipid-related synthases have been well researched for decades. SQDG synthases present a high homology between plants and microalgae. UDP-sulfoquinovose synthase (EC: 3.13.1.1) (SQD1) and SQDG synthase (EC: 2.4.1.-) (SQD2) are involved in the SQDG synthesis in both plants and eukaryotic microalgae [19,20]. In cyanobacteria, sqdB (EC: 3.13.1.1) and sqdX (EC: 2.4.1.-), responsible for SQDG synthesis [21,22], show high sequence similarity with SQD1 and SQD2, respectively [23]. However, genetic differences exist between MGDG and DGDG synthases in plants and microalgae. In plants, three MGDG synthases (EC: 2.4.1.46) (MGD1, MGD2 and MGD3) [24,25,26] and two DGDG synthases (EC: 2.4.1.241) (DGD1 and DGD2) [27,28] have been identified. MGD1 and DGD1 participate in the synthesis of the bulk of MGDG and DGDG, respectively, while DGD2 is involved in DGDG synthesis under specific growth conditions [28], with MGD2 and MGD3 providing MGDG as a precursor [29]. In *Chlamydomonas reinhardtii*, only orthologues of MGD1 and DGD1 were identified [20,30], but a second isoform of the DGDG synthase, resembling the plant DGD2, was additionally found in *Ostreococcus tauri* [20]. In cyanobacteria, no homolog for the plant-type DGDG synthase has been detected [31], and dgdA (EC: 2.4.1.241) (the DGDG synthase in cyanobacteria) is only distantly related to DGD1 [23,32]. MGDG synthesis in cyanobacteria is more complicated. All cyanobacteria are likely to synthesize MGDG through the epimerization of monoglucosyl diacylglycerol (MGlcDG) [33] by the MGlcDG synthase (EC: 2.4.1.336) (mgdA) [34,35] and the MGlcDG epimerase (EC 5.1.3.34) (mgdE) [36]. Some studies have reported that phosphate deficiency would stimulate the expression of glyceroglycolipid-related synthases [17,37,38,39], but the underlying regulation mechanism is still poorly understood. 

Metabolic engineering could modify the metabolisms of an organism so as to produce specific metabolites. According to recent reports, overexpressing a bHLH transcription factor [40] and a bZIP transcription factor [41] could enhance biomass and lipid productivity in *Nannochloropsis salina*. Overexpressing a soybean transcription factor, GmDof4, significantly enhanced the lipid production in *Chlorella ellipsoidea*, without sacrificing biomass [42]. The available information regarding the key regulators involved in cyanobacteria glyceroglycolipid metabolism is currently still deficient, which limits the development of cyanobacteria glyceroglycolipids utilization.

Previous studies in our lab indicated a decrease in MGDG and increases in DGDG and SQDG in *Synechococcus* sp. under phosphate starvation [2]. To investigate the relationship between phosphate starvation and glyceroglycolipid metabolism, the transcriptome of the cyanobacteria model organism *Synechococcus elongatus* PCC 7942 under phosphate starvation was analyzed in this study, which provided further insights into glyceroglycolipid metabolism under phosphate starvation, and a scientific basis for cyanobacteria glyceroglycolipids utilization in metabolic engineering.

## 2. Results and Discussion

### 2.1. Changes in Glyceroglycolipid Composition in Synechococcus elongatus PCC 7942 under Phosphate Starvation

**Figure 1:**
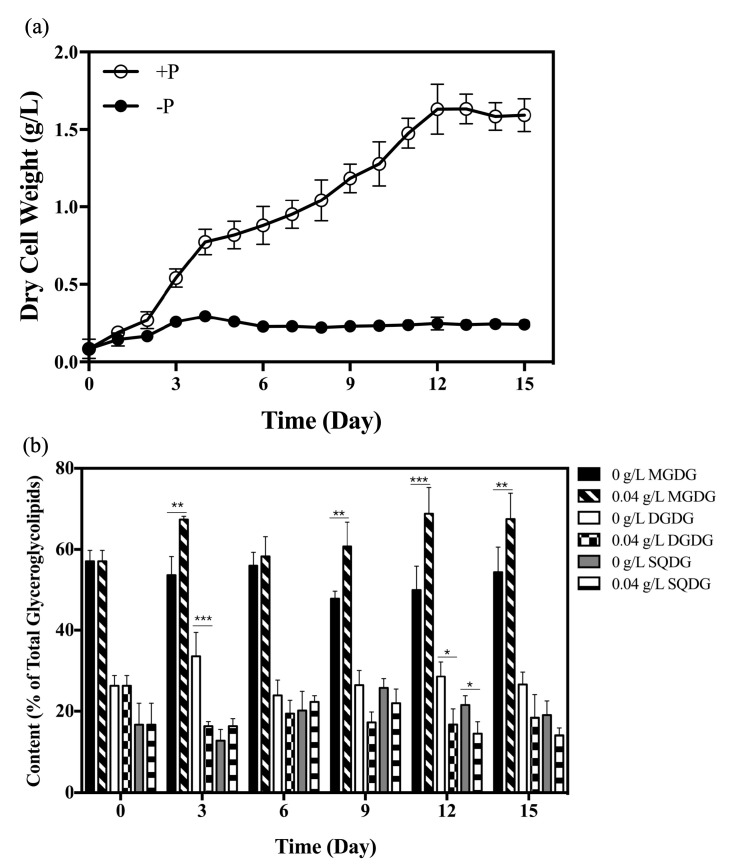
Growth (**a**) and dynamic changes in glyceroglycolipid composition (**b**) in *S. elongatus* PCC 7942 under phosphate concentrations of 0.04 g/L (+P) and 0 g/L (−P). Values are the means ± standard deviations from the three separately grown cultures. 2-way ANOVA test (* *p* < 0.05, ** *p* < 0.01, *** *p* < 0.001).

Our previous research demonstrated that the content of total glyceroglycolipids increased over all growth stages, and glyceroglycolipid composition changed in *S. elongatus* PCC 7942, under phosphate starvation, which helps cyanobacteria adapt to unfavorable conditions [43]. In order to gain more insight into glyceroglycolipid changes triggered by phosphate starvation, the growth, and dynamic variations in the composition, of three different glyceroglycolipids in *S. elongatus* PCC 7942 were investigated under the initial phosphate concentrations of 0.04 g/L and 0 g/L (Figure 1).

The growth of *S. elongatus* PCC 7942 under phosphate concentrations of 0.04 g/L reached a stationary phase after 12 days of cultivation (Figure 1a). The maximum biomass concentrations, with 0.04 g/L and 0 g/L phosphate, were 1.63 g/L and 0.25 g/L, respectively.

Under phosphate starvation, the difference in MGDG content was significant on day 3 (*p* = 0.0044), day 9 (*p* = 0.0067), day 12 (*p* = 0.0004) and day 15 (*p* = 0.006). The difference in DGDG content was significant on day 3 (*p* = 0.0009) and day 12 (*p* = 0.0145) under phosphate starvation. The difference in SQDG content was significant on day 12 (*p* = 0.0327). The largest difference in the composition of glyceroglycolipids in *S. elongatus* PCC 7942, caused by phosphate starvation, was observed on day 12 (Figure 1b). The MGDG content was 50% of the total glyceroglycolipids under phosphate starvation on day 12, which was 0.72 times lower than that in the phosphate-rich culture (69% of total glyceroglycolipids) (Figure 1b). DGDG and SQDG contents were 29% and 21% of total glyceroglycolipids, respectively, under phosphate starvation on day 12, which were respectively 1.70 and 1.50 times higher than that in the phosphate-rich culture (17% and 14% of total glyceroglycolipids) (Figure 1b), respectively. Many studies have already illustrated that phosphate starvation or limitation would cause lipid remodeling in many species, and found that the increased glyceroglycolipids could functionally substitute the degrading phospholipids [13,17,44]. This result demonstrated that the increase of total glyceroglycolipids content in *S. elongatus* PCC 7942 (Supplementary Figure S1) mainly resulted from the accumulation of DGDG and SQDG under phosphate starvation (Figure 1b), the same as *Synechocystis* sp. PCC 6803 [31], indicating their importance in adapting to phosphate stress [43]. 

### 2.2. Expressions of Glyceroglycolipid Synthase Genes in Synechococcus elongatus PCC 7942 under Different Phosphate Concentrations

To explore the changes in glyceroglycolipid composition at the transcriptional level, the expressions of glyceroglycolipid synthase genes in *S. elongatus* PCC 7942, cultivated under the initial phosphate concentrations of 0.04 g/L and 0 g/L for 12 days, were determined by qRT-PCR. In *S. elongatus* PCC 7942, *mgdA* (Synpcc7942_1083), *mgdE* (Synpcc7942_0861), *dgdA* (Synpcc7942_0986), *sqdB* (Synpcc7942_0578) and *sqdX* (Synpcc7942_0579) are the five glyceroglycolipid synthase genes. 

**Figure 2:**
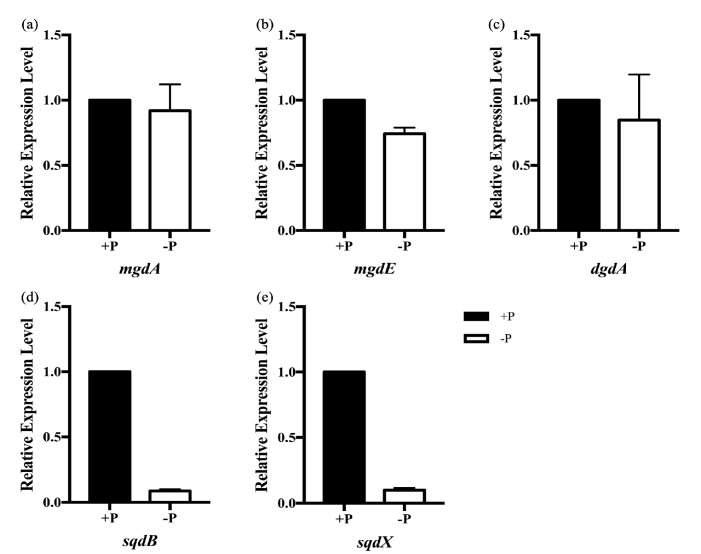
Relative gene expressions of *mgdA* (**a**), *mgdE* (**b**), *dgdA* (**c**), *sqdB* (**d**) and *sqdX* (**e**) in *S. elongatus* PCC 7942 cultivated under phosphate concentrations of 0.04 g/L (+P) and 0 g/L (−P) for 12 days. Values are the means ± standard deviations from the three separately grown cultures. The expression level of each glyceroglycolipid synthase gene under phosphate concentration of 0.04 g/L (+P) was set to 1.

The expression levels of *mgdA* and *mgdE* showed no significant differences under phosphate starvation (Figure 2a,b). The expression of *mdgA* is not regulated by the SphS–SphR two component system in response to inorganic phosphate [45]. The mgdA–mgdE system in cyanobacteria was replaced by MGD1 in eukaryotic microalgae and plants [33]. In *Arabidopsis*, the expression of *MGD1* is not induced by phosphate deficiency [29]. Moreover, the expression of *mdgA* also showed no differences under heat stress [46]. Thus, the mgdA–mgdE system may not be regulated at the transcriptional level.

As mentioned above, the increased DGDG accounted for the majority of the increased total glyceroglycolipids, and DGDG levels were increased throughout the whole culture period (Figure 1b and Supplementary Figure S2). However, the expression level of *dgdA* showed no significant differences under phosphate starvation (Figure 2c). In plants, both *DGD1* and *DGD2* can be up-regulated under conditions of phosphate deficiency [47]. This result suggested that the regulation of DGDG synthases in cyanobacteria might be different from that in plants, since an evolutionary gap exists between DGDG synthases in plants and cyanobacteria [31]. 

SQDG has been regarded as a surrogate for phosphatidylglycerol (PG), and SQDG synthases will be specifically induced upon phosphate starvation [1,17]. Interestingly, the expression levels of *sqdB* and *sqdX* were down-regulated by 90% (Figure 2d,e). The degrees of down-regulation in *sqdB* and *sqdX* are comparable, since *sqdB* and *sqdX* are likely to form an operon called *sqdBX* [48]. In this study, SQDG content was increased under phosphate starvation on day 12. It showed a slightly decreased trend after 12 days of cultivation, although this was not statistically significant (Supplementary Figure S2). 

### 2.3. Global Transcriptomic Analysis under Different Phosphate Concentrations

**Figure 3:**
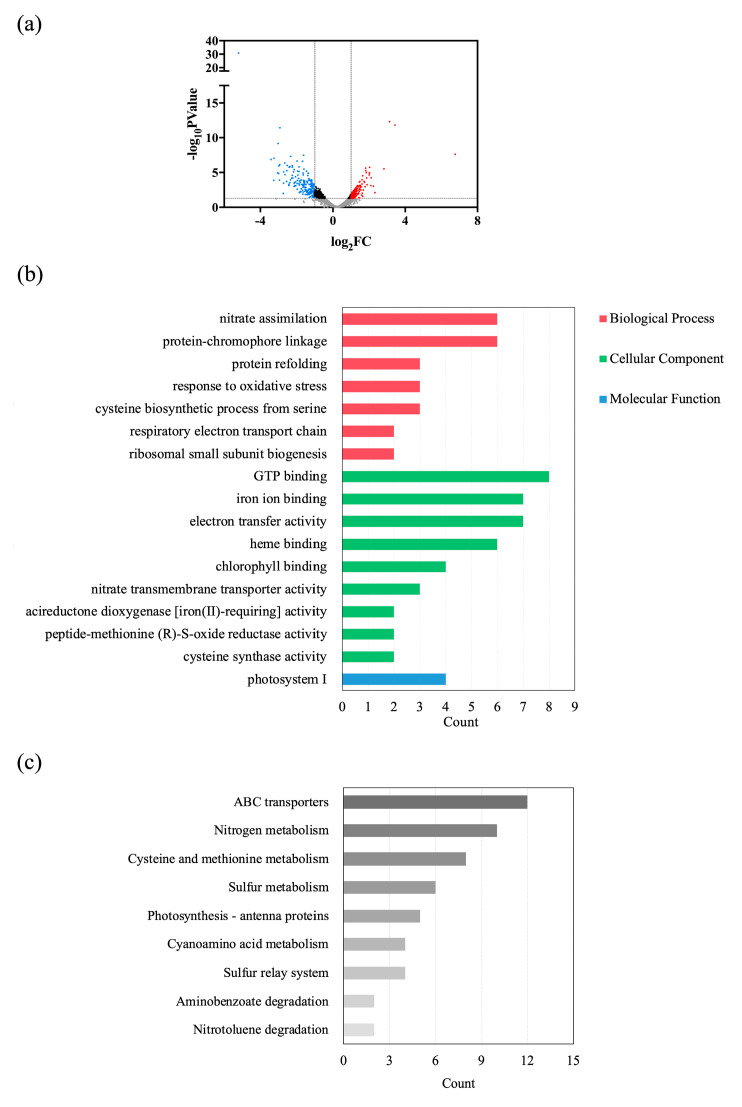
DEGs, and GO and KEGG enrichment of DEGs under phosphate starvation in *S. elongatus* PCC 7942. All DEGs were defined under *p*-value < 0.05 and fold change values (FC) ≥ 2 or ≤ 0.5. (**a**) Volcano plot of DEGs. Red and blue represent up-regulated and down-regulated DEGs, respectively. (**b**) Representative enriched GO terms of DEGs. Bars represent number of DEGs. (**c**) Representative enriched pathways of DEGs. Bars represent number of DEGs.

To further investigate the regulatory mechanism of changes in glyceroglycolipid composition, the transcriptome of *S. elongatus* PCC 7942, cultivated under the initial phosphate concentrations of 0.04 g/L and 0 g/L, for 12 days, was analyzed. As shown in Figure 3a and Supplementary Table S1, 2,660 genes in total were analyzed, among which 165 genes (6.2%) were significantly up-regulated and 172 genes (6.5%) were significantly down-regulated under phosphate starvation conditions, compared with the levels under 0.04 g/L phosphate concentration. 

The Gene Ontology (GO) enrichment of differentially expressed genes (DEGs), illustrated in Figure 3b and Supplementary Table S2, significantly enriched 17 GO terms. The most enriched GO term was GTP binding (Figure 3b), in which three DEGs were significantly down-regulated and five DEGs were significantly up-regulated (Supplementary Table S3), indicating the active signals transmitting under phosphate starvation. Notably, a gene coding the *Escherichia coli* Ras-like protein (*era*, Synpcc7942_0160) was significantly up-regulated, with a fold change value of 2.42 under phosphate starvation (Supplementary Table S3). In *S. elongatus* PCC 7942, an *era* overexpression strain exhibited significantly higher amounts of fatty acids compared to wild type [49]. Protein Era is highly conserved [50], and *ERA-related GTPase* (*ERG*) in plants is always related to chloroplast biogenesis [51,52,53], revealing its homologous function with cyanobacteria. Depletion of an Era-type GTP-binding protein resulted in abnormal chloroplasts lacking thylakoid membranes in rice, which indicated its importance in chloroplast development [54]. It could be speculated from this evidence in the literature that the accumulation of total glyceroglycolipids under phosphate starvation may be associated with the up-regulation of *era*. 

The other two highly enriched GO terms were iron ion binding and electron transfer activity, suggesting electron transport was severely affected after 12 days of cultivation under phosphate starvation. The Kyoto Encyclopedia of Genes and Genomes (KEGG) enrichment of DEGs (Figure 3c and Supplementary Table S4) showed that nine metabolic pathways were significantly affected by phosphate starvation, of which the three most enriched were ABC transporters, nitrogen metabolism, and cysteine and methionine metabolism. Similar responses were commonly implicated when organisms were exposed to adverse conditions [55,56].

Besides, GTP-binding proteins play important roles in the cell cycle, cell division and ribosome maturation [57]. We analyzed the DEGs involved in the cell cycle, cell division and ribosome maturation. According to GO enrichment (Supplementary Table S2) and KEGG enrichment (Supplementary Table S4), these metabolisms were not significantly enriched (*p*-value > 0.05) under conditions of phosphate starvation on day 12, though one gene involved in the cell cycle, two genes involved in cell division and nine genes involved in ribosome maturation were differentially expressed under phosphate starvation.

### 2.4. Differential Expressions of Genes Involved in Glyceroglycolipid Synthesis

**Figure 4:**
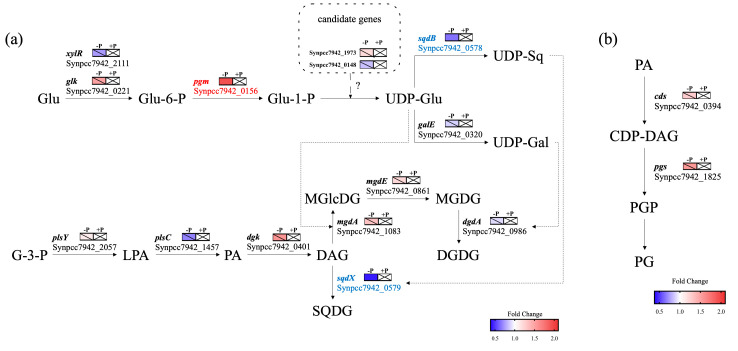
Metabolic pathway of glyceroglycolipid synthesis (**a**) and phospholipid synthesis (**b**) in *Synechococcus elongatus* PCC 7942. Pathways were reconstructed based on the KEGG annotation and the gene expression (colored rectangles) derived from transcriptome data of *S. elongatus* PCC 7942 (*p*-value < 0.05). Control groups (+P) are represented by rectangles with crosses. Genes up-regulated under phosphate starvation are indicated in red. Genes down-regulated are indicated in blue. Genes with no significant changes are indicated in black with diagonal lines in rectangles. *xylR*: xylose repressor (EC: 2.7.1.2); *glk*: glucokinase (EC: 2.7.1.2); *pgm*: phosphoglucomutase (EC: 5.4.2.2); *galE*: UDP-galactose epimerase (EC: 5.1.3.2); *sqdB*: UDP-sulfoquinovose synthase (EC: 3.13.1.1); *sqdX*: SQDG synthase (EC: 2.4.1.-); *plsY*: acyl-phosphate glycerol-3-phosphate acyltransferase (EC: 2.3.1.275); *plsC*: 1-acyl-sn-glycerol-3-phosphate acyltransferase (EC: 2.3.1.51); *dgk*: diacylglycerol kinase (EC: 2.7.1.107); *mgdA*: MGlcDG synthase (EC: 2.4.1.336); *mgdE*: MGlcDG epimerase (EC: 5.1.3.34); *dgdA*: DGDG synthase (EC: 2.4.1.241); *cds*: phosphatidate cytidylyltransferase (EC: 2.7.7.41); *pgs*: CDP-diacylglycerol-glycerol-3-phosphate 3-phosphatidyltransferase (EC: 2.7.8.5). Glu: glucose; Glu-6-P: glucose-6-phosphate; Glu-1-P: glucose-6-phosphate; UDP-Glu: UDP-glucose; UDP-Gal: UDP-galactose; UDP-Sq: UDP-sulfoquinovose; G-3-P: glycerol-3-phosphate; LPA: Lysophosphatidic acid; PA: phosphatidic acid; DAG: diacylglycerol; CDP-DAG: CDP-diacylglycerol; PGP: phosphatidylglycerophosphate; PG: phosphatidylglycerol; MGlcDG: monoglucosyl diacylglycerol; MGDG: monogalactosyl diacylglycerol; DGDG: digalactosyl diacylglycerol; SQDG: sulfoquinovosyl diacylglycerol.

The glyceroglycolipid synthesis pathway, based on KEGG annotation, is shown in Figure 4a. In total, 12 genes were involved in this pathway. However, the UDP-glucose pyrophosphorylase (EC: 2.7.7.9) (ugp) responsible for transforming glucose-1-phosphate to UDP-glucose remained unidentified. In *S. elongatus* PCC 7942, a conserved hypothetical protein coded by Synpcc7942_0148 showed a percent identity of 49% with the ugp in *Synechocystis* sp. PCC 6803, coded by *slr0207* [58], indicating Synpcc7942_0148 is possibly responsible for UDP-glucose synthesis. Moreover, the unique cyanobacteria UDP-glucose pyrophosphorylase (cugP), coded by *sll1558*, which is annotated as mannose-1-phosphate guanyltransferase (EC:2.7.7.13) (GMPP) but displays ugp activity, was identified in *Synechocystis* sp. PCC 6803 [59]. In *S. elongatus* PCC 7942, GMPP coded by Synpcc7942_1973 showed a percent identity of 79% with the product of *sll1558*, suggesting it is involved in UDP-glucose synthesis as a cugP.

According to transcriptome analysis, *mgdA, mgdE* and *dgdA* showed no significant differences in expression under conditions of phosphate starvation, while *sqdB* and *sqdX* were down-regulated, with fold changes of 0.63 and 0.43, respectively (Figure 4a and Supplementary Table S5), the same as the results of qRT-PCR. Moreover, the expressions of other genes related to glyceroglycolipid synthesis, and the two possible candidates responsible for UDP-glucose synthesis (Synpcc7942_0148 and Synpcc7942_1973), showed no significant differences under phosphate starvation as well (Figure 4a), except for *pgm* (Synpcc7942_0156), which is responsible for transforming glucose-6-phosphate into glucose-1-phosphate, and which was up-regulated with a fold change of 1.93 (Figure 4a and Supplementary Table S5). The up-regulation of *pgm* will lead to an accumulation of glucose-1-phosphate, the precursor of UDP-glucose, which provides glycosyl for glyceroglycolipid synthesis, thus accounting for the increase of glyceroglycolipids. 

Besides the de novo synthesis of glyceroglycolipids, phosphate starvation could directly influence phospholipid degradation through phospholipases [17,44,60] to provide precursors of glyceroglycolipids. The phosphatidic acid (PA) produced by type D phospholipases (PLDs) would also be a candidate in the activation of MGDG synthesis, acting as a signal molecule [61]. Unlike various phospholipids existing in plants, eukaryotic microalgae and other photosynthetic organisms, PG is the only phospholipid in the thylakoid and cytoplasmic membranes of cyanobacteria [62], but the phosphatidylglycerophosphatase (EC: 3.1.3.27) (pgp) responsible for PG synthesis remains unidentified in most cyanobacteria. Whereas PG was degraded by phosphate starvation in our previous study [43], no significant differences were shown in the expressions of the two annotated genes related to the PG synthesis (Figure 4b) and Synpcc7942_0302 coding of the only identified PLD (Supplementary Table S5). In *Nannochloropsis oceanica*, phospholipid degradation mainly resulted from the up-regulation of type A phospholipases (PLAs) under conditions of phosphate limitation, since PLD genes were suppressed [17], although phosphatidylcholine (PC) hydrolyzation by PLDs is a main strategy for phospholipid degradation under cold stress [63]. In *Synechocystis* PCC6803, the remolding of PG involves reactions catalyzed by phospholipases A1 and A2, although the phospholipases have not yet been identified [64]. As yet, no homologs of PLA have been identified in *S. elongatus* PCC 7942.

The high catalytic rates of related enzymes may be a possible cause of this phenomenon. Besides, not all glyceroglycolipid-related genes respond to phosphate deficiency at the transcriptional level [29]. Meanwhile, the evidence in the literatures suggests that galactolipid synthesis in plants is not only modified at the transcriptional level, but also at the post-transcriptional [65] or post-translational level [61,66]. Selão et al. also demonstrated that lipid-synthesizing enzymes in both *Synechococcus* and *Synechocystis* must be regulated at the post-translational level, by temperature, rather than at the transcriptional level [45]. These results suggest that a more complicated regulatory mechanism exists in glyceroglycolipid synthesis.

### 2.5. Glyceroglycolipid Homeostasis in Glyceroglycolipid Synthesis under Phosphate Starvation

Microalgae, cyanobacteria and plants all possess mechanisms for establishing lipid homeostasis in thylakoid membranes [1]. The overexpressions of *sqdB* and *sqdX* would lead to up-regulations of *mgdA*, *mgdE* and *dgdA*, so as to maintain lipid homeostasis [67]. In this study, because DGDG increased and MGDG decreased under phosphate starvation, the MGDG/DGDG ratio fell to 5:3 on day 12 (4:1 in the phosphate-rich culture) (Figure 1b). This result was similar to the result for *M. subterraneus*, in which the MGDG/DGDG ratio was 2:1 in the control culture, which then decreased to about 1:1 under P-deprivation [68]. The MGDG/DGDG ratio appears stable under favorable controlled conditions [1], and it is crucial for the physical state of chloroplast membranes [68]. The control of the MGDG/DGDG ratio is a main feature of thylakoid lipid homeostasis [1]. Because of the decrease in the MGDG/DGDG ratio under phosphate starvation, more MGDG had to be synthesized in order to maintain the original MGDG/DGDG ratio. Besides, MGDG itself plays an important role in the proper development of thylakoid membranes in plants [65,69], and the synthesis of MGDG can be activated by lipid molecules (like PA, SQDG and PG) [45,66,70], indicating the importance of MGDG regulation in maintaining lipid homeostasis.

The expressions of *sqdB* and *sqdX* were down-regulated after 12 days of cultivation (Figure 2d,e and Figure 4a). It could be inferred that this phenomenon was associated with lipid homeostasis regulation. Because MGDG and SQDG are synthesized with the same precursors (diacylglycerol and UDP-glucose), the down-regulation of *sqdB* and *sqd*X could result in more precursors participating in MGDG synthesis. Besides, the down-regulation of *sqdB* and *sqdX* could also result in more precursors being available for DGDG synthesis. However, unlike DGDG synthesis, which is more strongly regulated at the transcriptional level, post-transcriptional and post-translational regulations are more important for MGDG synthesis in plants [65]. The increased level of SQDG under conditions of phosphate starvation could activate the activity of mgdA [45]. Thus, the available precursors provided by the down-regulation of *sqdB* and *sqdX* are likely to contribute more to MGDG synthesis. Moreover, the up-regulation of *pgm* (Figure 4a) could also provide an adequate precursor for MGDG synthesis. Under phosphate starvation, the MGDG/DGDG ratio increased back to 2:1 after 15 days of cultivation (Figure 1b). Thus, the down-regulation of *sqdB* and *sqdX*, together with the increased level of SQDG, contributed to the activating of MGDG synthesis under phosphate starvation.

Moreover, MGDG, DGDG and SQDG are essential for maintaining the stability of the photosystem [71,72]. Our previous study showed that the functions of the photosystem were nearly damaged when exposed to phosphate stress [43]. In this study, genes related to photosynthesis were repressed (Supplementary Table S6) by phosphate starvation. In plants, galactolipid biosyntheses are coordinated with photosynthetic protein synthesis [66]. Thus, the photosynthetic apparatus was likely to be disrupted, in part, by changes in the MGDG/DGDG ratio resulting from phosphate starvation, and a proper ratio of thylakoid membrane lipids was needed to help the photosynthetic apparatus recover.

In summary, in order to maintain the integrity of photosynthetic membranes and the photosynthetic apparatus, the regulation of glyceroglycolipid composition is based on the MGDG/DGDG ratio, which helps *S. elongatus* PCC 7942 maintain resiliency when exposed to favorable conditions in culture.

### 2.6. Regulatory Networks Involved in Glyceroglycolipid Synthesis

In plants and eukaryotic microalgae, some lipid-related transcription factors, like Dofs [73], are glyceroglycolipid-related. Nevertheless, transcriptional regulation is quite different between eukaryotes and prokaryotes. To determine the transcriptional regulators related to glyceroglycolipid metabolism under conditions of phosphate starvation in *S. elongatus* PCC 7942, 12 differentially expressed transcription regulators were selected, of which six were up-regulated and six were down-regulated (Table 1). These 12 transcription regulators can be divided into eight types: MarR family (1), MerR family (1), two component system (4), ArsR family (1), BadM/Rrf2 family (1), XRE family (2), GntR family (1) and DevT-like transcriptional factor (1). Most of these differentially expressed transcription regulators are involved in responses to various environmental stress conditions.

However, the genes coding the SphS–SphR phosphate sensing system and genes regulated by the system [74] showed no significant differences on day 12 of phosphate starvation, suggesting that these genes may respond to phosphate starvation at an early stage. Interestingly, an OmpR family response regulator gene *nblR* (Synpcc7942_2305), which regulates the degradation of phycobilisome (PBS) through the *nbl* pathway, as an activator of the PBS degradation protein gene’s (*nblA*) transcription under stresses [75], was down-regulated under phosphate starvation. However, *nblA* (Synpcc7942_2127) was significantly up-regulated (Supplementary Table S1), indicating that the down-regulation of *nblR* on day 12 was not to regulate PBS degradation. Sato et al. inferred that the *sqdB* might be involved in S-starvation-induced PBS degradation, particularly in *Synechococcus* [48]. It could be supposed that *sqdB* somehow belongs to the *nbl* pathway, and *nblR* is a probable signaling component in *sqdBX* regulation. In addition, some photosynthesis-associated transcription factors, like HY5 (a basic Leu zipper transcription factor) and GOLDEN2-LIKE (GLK), play pivotal roles in plant glyceroglycolipid regulation [66]. Therefore, glyceroglycolipid metabolism and the formation of photosynthetic machineries may be affected mutually. Besides, the up-regulated XRE family transcription regulator gene Synpcc7942_0110 was probably a hub gene under phosphate starvation, according to the protein–protein interaction analysis of DEGs (Supplementary Figure S3), which indicated that Synpcc7942_0110 played an important role in the global regulation of *S. elongatus* PCC 7942 under phosphate starvation.

The post-transcriptional and post-translational regulations are of great significance to glyceroglycolipid metabolism in plants. In the detached cotyledons of cucumber with impaired *csMGD1* expression, light may activate MGDG biosynthesis in a post-transcriptional manner [76]. According to transcriptome analysis, three genes involved in sulfur relay system were significantly up-regulated (Supplementary Tables S1 and S4), suggesting that active tRNA modification under phosphate starvation contributed to metabolic regulation [77,78]. The activities of MGDG synthases can be modified by thioredoxins at the post-translational level [66]. In this study, a thioredoxin reductase gene (Synpcc7942_0623), a thioredoxin gene (Synpcc7942_1793) and a thioredoxin peroxidase gene (Synpcc7942_2309) were significantly up-regulated (Supplementary Table S1), all of which may contribute to activating MGDG synthesis. Like SQDG, PA and PG also play important roles in activating MGD1 [66,70]. Moreover, PG contributes to inducing MGDG synthesis by anchoring MGD1 and bringing substrates closer to the active site [66]. The information concerning the post-transcriptional and post-translational regulations involved in the glyceroglycolipid metabolism of cyanobacteria is still deficient, and thus more future studies are needed. In plants, auxin and cytokinin act as mediums between environmental conditions and the glyceroglycolipid metabolism [47,76]. Kobayashi et al. demonstrated that changes in plant membrane lipids during phosphate starvation are regulated by Pi signaling and auxin/cytokinin cross-talk [47]. In *S. elongatus* PCC 7942, increased SQDG, induced by overexpressions of *sqdB* and *sqdX*, would result in the abnormal expression of cell division-related genes and abnormal cell division [67]. The cell division protein gene *FtsQ* (Synpcc7942_2377) and the GroES protein gene (Synpcc7942_2314) involved in cell division were also important in global regulation under conditions of phosphate starvation (Supplementary Figure S3). Glyceroglycolipid synthesis and cell division could interact with each other. Taken together, it could be inferred that the glyceroglycolipid metabolism in cyanobacteria, under phosphate starvation, is regulated not only by Pi signaling, but also by other types of signaling and other metabolic pathways as well.

### 2.7. qRT-PCR Confirmations of Differentially Expressed Transcripts

qRT-PCR was used to confirm the accuracy of the transcriptomic analysis and measure the relative expression of selected transcripts. The results in Table 2 show that 82.4% of the measured transcripts (14 out of 17) followed the same trend as the RNA-Seq data, except for Synpcc7942_2416, Synpcc7942_1897 and Synpcc7942_1725. According to Celine, E. et al. [79], because of technical differences, over 80% of the measured genes having concordant expression represents a high concordance between RT-qPCR and RNA-seq. The expression of DEGs in transcriptomic analysis was reliable.

### 2.8. Reconstruction of Putative Glyceroglycolipid Regulatory Networks Based on Transcriptomic Evidence

**Figure 5:**
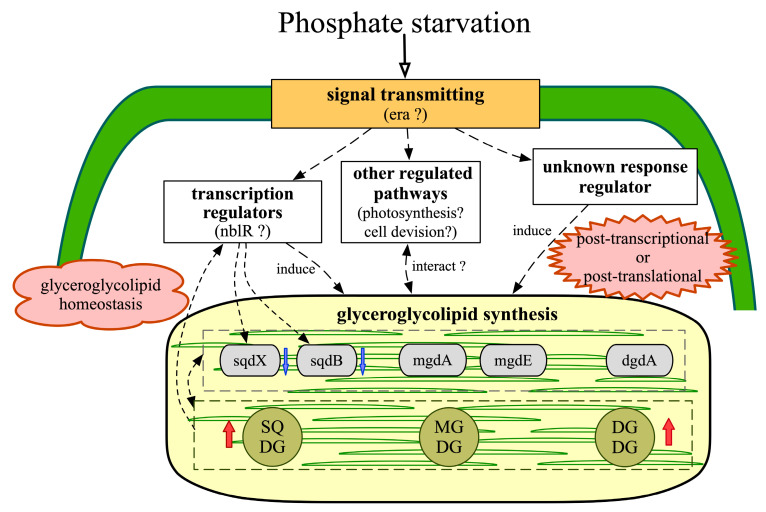
Putative model for glyceroglycolipid metabolism in *S. elongatus* PCC 7942 under phosphate starvation, reconstructed from transcriptomic evidence in this study. Dotted arrows indicate the regulation between each part.

Based on the transcriptomic evidence, a putative model for the glyceroglycolipids metabolism in *S. elongatus* PCC 7942, under phosphate starvation, was reconstructed (Figure 5).

Under phosphate starvation, the signals transmitting through GTP-binding proteins are active. The increase in total glyceroglycolipids mainly results from the accumulation of DGDG and SQDG as functional substitutes for phospholipids under phosphate starvation [43]. However, the changes in the composition of total glyceroglycolipids result in a sharp decrease of the MGDG/DGDG ratio, which disrupts the normal function of the plastid membrane [1]. To maintain glyceroglycolipid homeostasis, the down-regulations of *sqdB* and *sqdX* result in more precursors participating in MGDG synthesis, and the increased SQDG could act as an activator to enhance the activity of mgdA [45]. Transcriptional regulation, together with post-transcriptional and post-translational regulations, comprise an efficient strategy for glyceroglycolipid synthesis. Besides, glyceroglycolipids synthesis could interact with other metabolic pathways [66,67]. 

Hence, it could be proposed that the changes in glyceroglycolipid composition are emergency strategies of *S. elongatus* PCC 7942 adapting to phosphate starvation, and after 12 days of cultivation, glyceroglycolipid homeostasis plays a more important role in the recovery from phosphate starvation. 

## 3. Materials and Methods 

### 3.1. Cyanobacteria Species and Treatments

The cyanobacteria species in this study is *Synechococcus elongatus* PCC 7942 provided by Prof. Dingji Shi (Shanghai Ocean University, Shanghai, China). 

*S. elongatus* PCC 7942 was cultivated in a 1-L Erlenmeyer flask with 500 mL working volume of modified BG-11 medium, under a temperature of 25 ± 2 °C and aeration rate of 140 µmol/m^2^/s. Standard BG-11 contains 0.04 g/L K_2_HPO_4_ concentration, while phosphate-starved BG-11 lacks K_2_HPO_4_. The light intensity was 8000 lx. The initial pH was 8.0.

The culture’s optical densities were measured at 730 nm by a UV-Vis spectrophotometer (Tianmei, Shanghai, China). Cell density was calculated with the equation: cell density (g/L) = 0.3349 × OD_730_ − 0.0129 (*R*^2^ = 0.9926). Standard curves are shown in Supplementary Material, Figure S4.

### 3.2. Glyceroglycolipid Analysis

Three different glyceroglycolipids were separated with a modified method of thin-layer chromatography (TLC) (Huanghai, Yantai, China) [43,80]. First, total lipids were extracted from cyanobacteria with a modified method [2,81]. Freeze-dried cyanobacteria powder (0.2 g) was broken by a cell crusher (Tissuelyser-24, Jingxin, Shanghai) at 50 Hz for 10 min. Then the broken cells were suspended in a 5-mL solvent mixture of chloroform/methanol (v/v 2:1). After being stirred for 20 min, the samples were centrifuged at 8000 rpm for 10 min. The procedure was repeated three times until the total lipids were fully extracted. The solvent phase was transferred and evaporated in a water bath (Shanghai YIHENG Technical Co. Ltd., Shanghai, China) at 65 °C and then dried in a drying oven (Jinghong, Shanghai, China) at 50 °C until the weight was stable. Then the total lipids were weighed with analytical balance (BS 124S, Sartorius, GöF6ttingen, Germany) and redissolved in chloroform/methanol (v/v 2:1) at a concentration of 10 µg/µL. The developing solvent was acetone/toluene/water (v/v/v 91:30:8). Different glyceroglycolipids were visualized in iodine vapor. 

For quantitative analysis, fatty acid methyl esters of each glyceroglycolipid were prepared with 2 mL anhydrous 1 N methanolic HCl, and then incubated at 80 °C for 30 min [82]. The fatty acid profiles of different glyceroglycolipids were analyzed by AutoSystem XL GC/TurboMass MS (Perkin Elmer, Rodgau, Germany) [81]. The internal control was nonadecanoic acid.

The methods of calculating the content of each glyceroglycolipid were based on the method of Benning [80]. A total of six samples (two for each of the three cultures) per strain were analyzed, and means and standard deviations were calculated.

### 3.3. RNA Extraction, Library Preparation and Sequencing

Cyanobacteria cells were harvested in triplicate by centrifugation on day 12 under the phosphate concentrations of 0 and 0.04 g/L, respectively. Cells were immediately transferred to liquid nitrogen for later processing. Total RNA of each harvested sample was extracted with TRIzol reagent (Invitrogen Life Technologies) according to the manufacturer’s protocol. The sequencing library of each harvested sample was generated using a TruSeq RNA Sample Preparation Kit (Illumina, San Diego, CA, USA). The library was then sequenced on a HiSeq platform (Illumina) by Shanghai GeneFund Biotechnology Co. Ltd.

### 3.4. Transcript Quantification and Differential Expression Analysis

The clean reads were obtained by trimming raw reads from the sequencing with a Cutadapt tool [83] and a Trimmomatic tool [84]. The quality of clean reads was also assessed via FastQC tool [85]. Then, the high-quality trimmed reads were mapped to the reference genome by Bowtie2 [86]. Gene expression data were obtained and quantified with the fragments per kilobase of exon per million reads mapped method (FPKM) by HTSeq [87]. Empirical analysis of Digital Gene Expression in R (EdgeR) was applied for differential expression analysis [88]. Genes with *p*-value < 0.05 and fold change values (FC) ≥ 2 or ≤ 0.5 were regarded as DEGs.

GO enrichment analysis and KEGG enrichment analysis of DEGs were performed based on the hypergeometric distribution [89]. All DEGs were mapped to each GO term [90] and KEGG pathway [91]. The GO terms and the KEGG pathways of DEGs with a *p*-value < 0.05 were considered significantly enriched.

### 3.5. Experimental Validation of Gene Expression with qRT-PCR

The synthesis of cDNA was performed using FastKing RT Kit (With gDNase) (TIANGEN, Beijing, China). The gene-specific quantitative real-time PCR primers used in this study were documented in Supplementary Table S7. Real-time PCR was performed using a SuperReal PreMix Plus (SYBR Green) (TIANGEN, Beijing, China), and was carried out using an Eppendorf Mcep Realplex 4s System (Eppendorf, Hamburg, Germany). Reactions started at 95 °C for 15 min, followed by 40 cycles of 95 °C for 10 s and 60 °C for 25 s, and a melting curve step at 60–95 °C. Each qRT-PCR reaction was performed on three biological replicates. The relative expression levels were normalized to the glyceraldehyde-3-phosphate dehydrogenase (GAPDH) gene (*gap3*, Synpcc7942_1939), RNA polymerase sigma factor gene (*rpoD*, Synpcc7942_0649) [92] and phosphoenolpyruvate carboxylase gene (*ppc*, Synpcc7942_2252) [93], and were calculated using the 2^-∆∆CT^ method [94]. 

## 4. Conclusions

The glyceroglycolipid composition of *S. elongatus* PCC 7942 changes to adapt to phosphate starvation. Glyceroglycolipid composition is regulated post-transcriptionally or post-translationally, allowing for more efficient adaptation to phosphate stress conditions. However, after 12 days of cultivation, the glyceroglycolipid composition is mainly regulated based on the MGDG/DGDG ratio in order to maintain the glyceroglycolipid homeostasis, which is beneficial in maintaining resilience when exposed to the preferred culture conditions again.

## Figures and Tables

**Table 1:** Differentially expressed transcription regulators under phosphate starvation. Up-regulated genes (FC > 1) and down-regulated genes (FC < 1) were ordered by FC values.

Gene ID	Annotation	FC	*p*-Value
Synpcc7942_0938	transcriptional regulator, ArsR family	8.79281803	5.0074E-13
Synpcc7942_2585	transcriptional regulator, BadM/Rrf2 family	2.43751415	0.00232243
Synpcc7942_2416	two component transcriptional regulator, winged helix family	2.15739652	0.04603509
Synpcc7942_0110	transcriptional regulator, XRE family	2.13386852	0.02139503
Synpcc7942_1897	putative transcription factor DevT-like	2.12534589	0.00718275
Synpcc7942_1725	transcriptional regulator, GntR family	1.94907168	0.04567919
Synpcc7942_2305	two component transcriptional regulator, winged helix family, nblR	0.72020702	0.04515754
Synpcc7942_1739	transcriptional regulator, MerR family	0.61129814	0.02357565
Synpcc7942_0556	two component transcriptional regulator, winged helix family	0.61086684	0.01166383
Synpcc7942_2466	two component transcriptional regulator, winged helix family	0.58625772	0.03952819
Synpcc7942_1159	transcriptional regulator, MarR family	0.57296615	0.0038297
Synpcc7942_0764	transcriptional regulator, XRE family	0.47965835	0.00047173

**Table 2:** qRT-PCR analysis of different genes under phosphate starvation in *Synechococcus elongatus* PCC 7942.

Gene ID	Annotation	FC	qRT-PCR
Synpcc7942_0938	transcriptional regulator, ArsR family	8.792818	2^3.18^
Synpcc7942_2585	transcriptional regulator, BadM/Rrf2 family	2.437514	2^3.82^
Synpcc7942_2416	two component transcriptional regulator, winged helix family	2.157397	2^−0.84^
Synpcc7942_0110	transcriptional regulator, XRE family	2.133869	2^2.78^
Synpcc7942_1897	putative transcription factor DevT-like	2.125346	2^−1.01^
Synpcc7942_1725	transcriptional regulator, GntR family	1.949072	2^−1.58^
Synpcc7942_1083	a probable glycosyltransferase, mgdA	1.33778	2^−0.14^
Synpcc7942_0861	a conserved hypothetical protein, mgdE	1.205566	2^−0.43^
Synpcc7942_0986	a probable glycosyltransferase, dgdA	0.90492	2^−0.32^
Synpcc7942_2305	two component transcriptional regulator, winged helix family	0.720207	2^−2.79^
Synpcc7942_0578	UDP-sulfoquinovose synthase, sqdB	0.634708	2^−3.54^
Synpcc7942_1739	transcriptional regulator, MerR family	0.611298	2^−3.70^
Synpcc7942_0556	two component transcriptional regulator, winged helix family	0.610867	2^−3.49^
Synpcc7942_2466	two component transcriptional regulator, winged helix family	0.586258	2^−4.39^
Synpcc7942_1159	transcriptional regulator, MarR family	0.572966	2^−4.38^
Synpcc7942_0764	transcriptional regulator, XRE family	0.479658	2^−1.02^
Synpcc7942_0579	sulfolipid sulfoquinovosyl diacylglycerol biosynthesis protein, sqdX	0.425591	2^−3.34^

## Supplementary Materials

The following are available online at https://www.mdpi.com/1660-3397/18/7/360/s1, Figure S1: Total glyceroglycolipids content under phosphate starvation. Values are the means ± standard deviations from the three separately grown cultures. Figure S2: Glycerolglycolipids relative content under phosphate starvation given by TLC-scanner. (a) MGDG. (b) DGDG. (c) SQDG. Values are the means ± standard deviations from the three separately grown cultures. Figure S3: PPI analysis of differentially expressed genes in *Synechococcus elongatus* PCC 7942 under different phosphate concentrations (0, 0.04g/L) after being cultivated for 12 days (Genes with annotation in String database are represented by abbreviation. Genes without annotation in String database are represented by gene number. Names of genes with degree less than 5 are not shown). Figure S4: Standard curve of OD_730_ and Dry Cell Weight of *Synechococcus elongatus* PCC 7942. Table S1: Differentially expressed genes under phosphate starvation in *Synechococcus elongatus* PCC 7942. Table S2: Enriched GO terms under phosphate starvation in *Synechococcus elongatus* PCC 7942. Table S3: Expression of genes in GO term GTP binding in *Synechococcus elongatus* PCC 7942 under phosphate starvation. Table S4: Enriched KEGG pathways under phosphate starvation in *Synechococcus elongatus* PCC 7942. Table S5: Expression of genes participating in glyceroglycolipid synthesis in *Synechococcus elongatus* PCC 7942 under phosphate starvation. Table S6: Expression of genes related to photosynthesis in *Synechococcus elongatus* PCC 7942 under phosphate starvation. Table S7: Primers used in qRT-PCR.
