# Supplementary material for: Glyceroglycolipid Metabolism Regulations under Phosphate Starvation Revealed by Transcriptome Analysis in Synechococcus elongatus PCC 7942
Source: Mar Drugs. 2020 Jul 13;18(7):360. doi: 10.3390/md18070360 (PMC7401256; doi:10.3390/md18070360)
Supplement: Supplementary file 1 [file marinedrugs-18-00360-s001.zip › Supplementary File-Revised/Supplementary file 1.docx]

**Supplementary file 1**


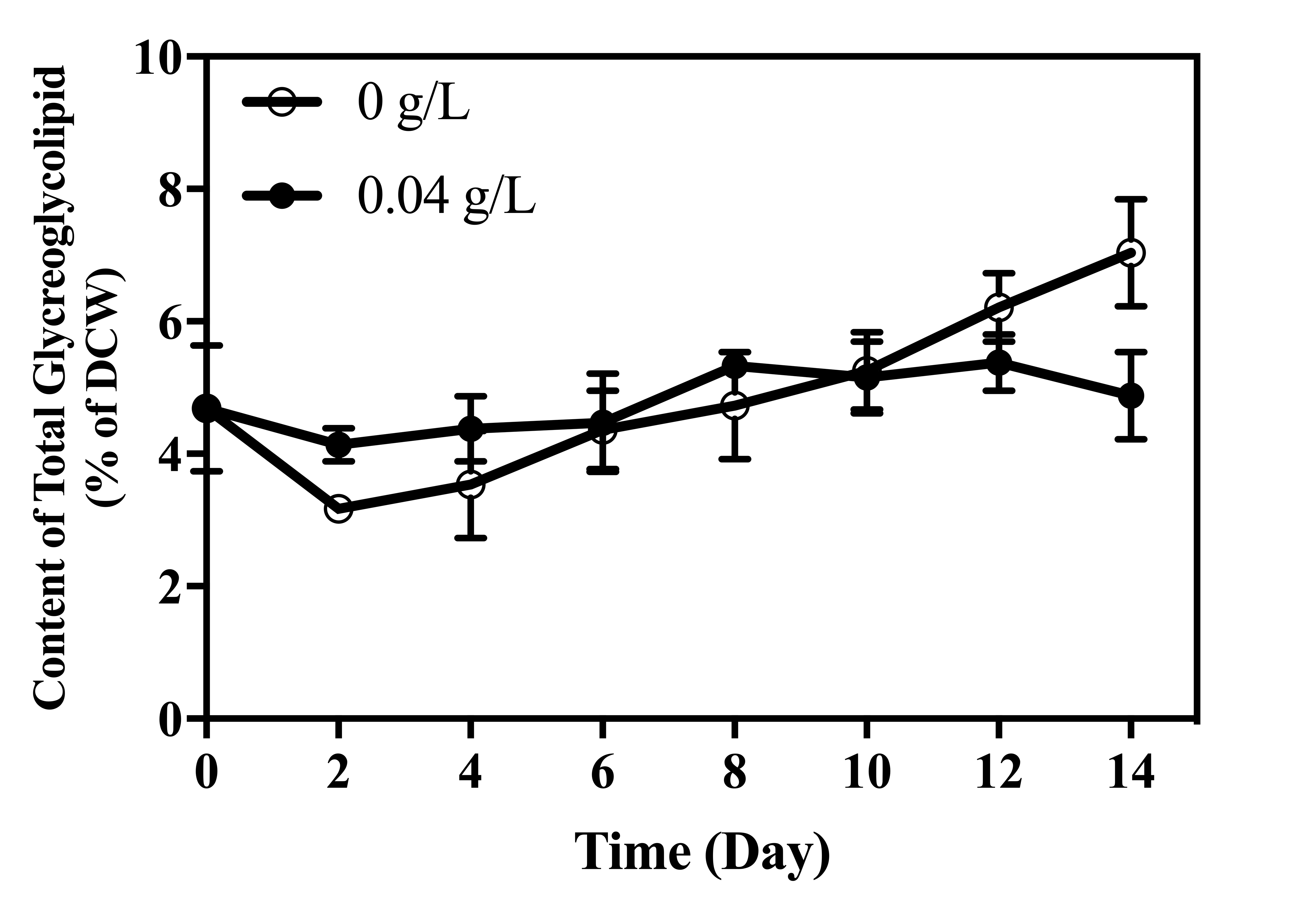


Supplementary Figure 1. Total glyceroglycolipids content under phosphate starvation. Values are the means ± standard deviations from the three separately grown cultures.


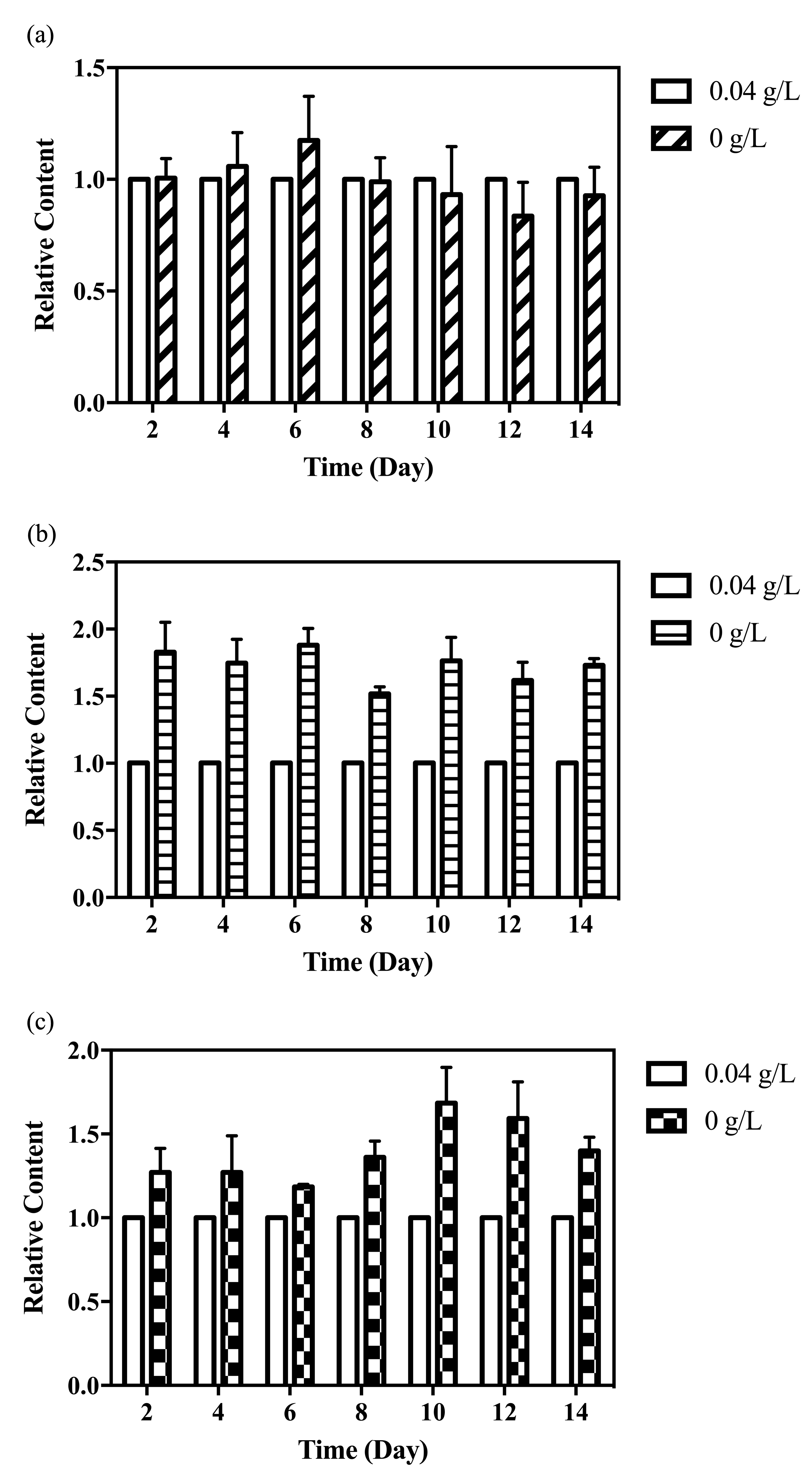


Supplementary Figure 2. Glycerolglycolipids relative content under phosphate starvation given by TLC-scanner. Values are the means ± standard deviations from the three separately grown cultures. (a) MGDG. (b) DGDG. (c) SQDG.


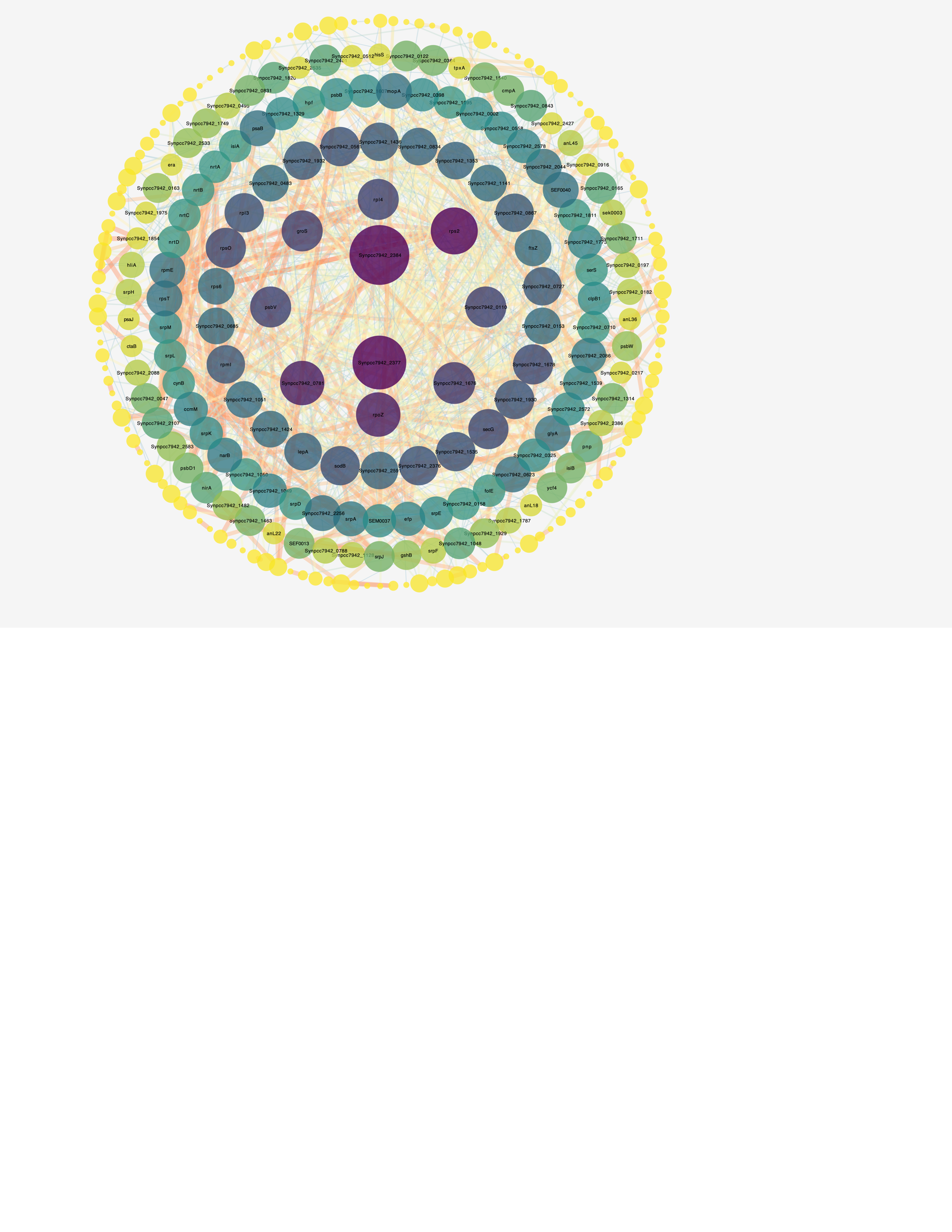


Supplementary Figure 3. PPI analysis of differentially expressed genes in *Synechococcus elongatus* PCC 7942 under different phosphate concentrations (0, 0.04g/L) after cultivated for 12 days (Genes with annotation in String database are represented by abbreviation. Genes without annotation in String database are represented by gene number. Names of genes with degree less than 5 are not shown).


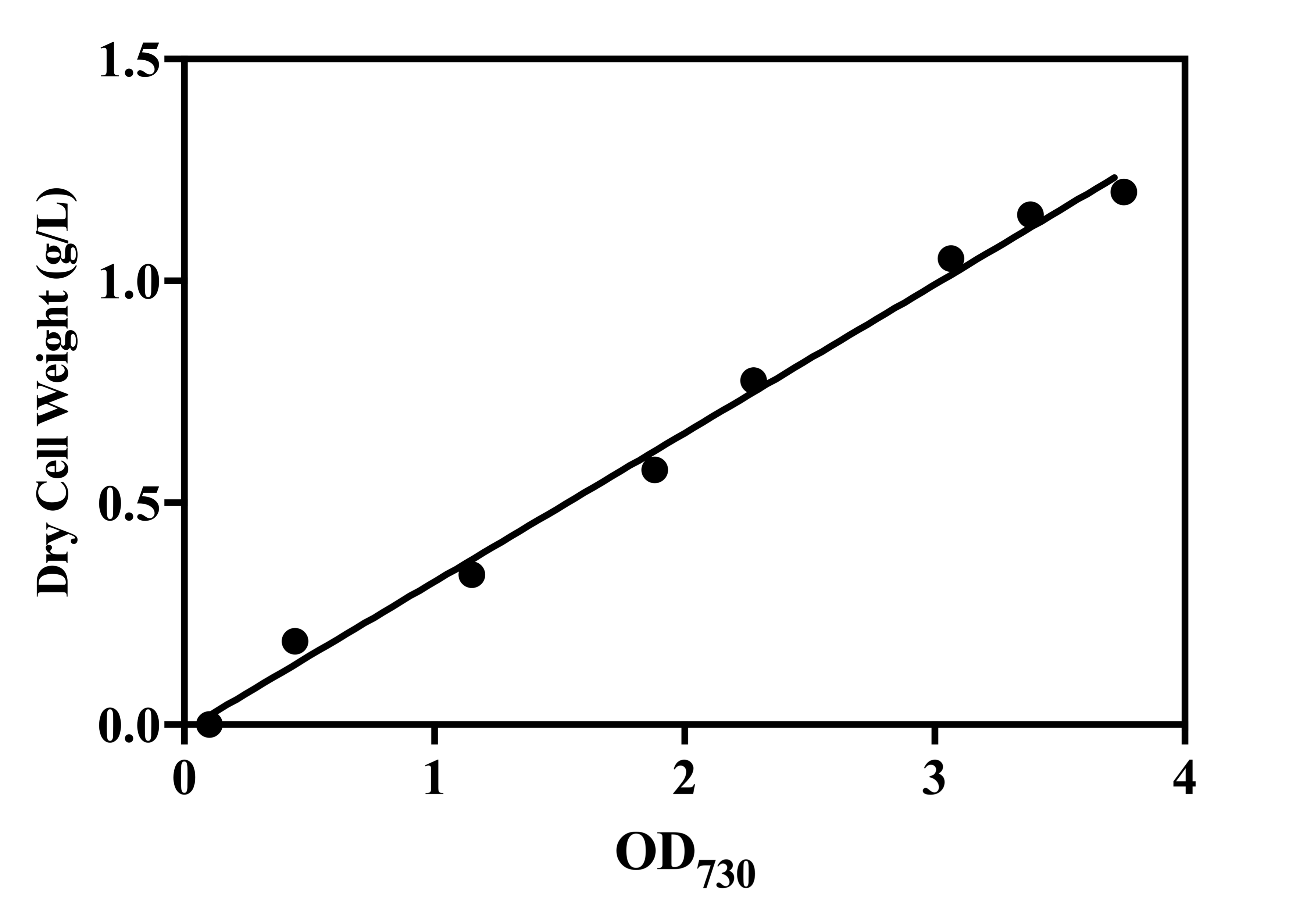


Supplementary Figure 4. Standard curve of OD_730_ and Dry Cell Weight of *Synechococcus elongatus* PCC 7942.
